# Investigation of Silk Fibroin/Poly(Acrylic Acid) Interactions in Aqueous Solution

**DOI:** 10.3390/polym16070936

**Published:** 2024-03-29

**Authors:** Jelena Škrbić, Ljiljana Spasojević, Altynay Sharipova, Saule Aidarova, Alpamys Babayev, Raziya Sarsembekova, Ljiljana Popović, Sandra Bučko, Jelena Milinković Budinčić, Jadranka Fraj, Lidija Petrović, Jaroslav Katona

**Affiliations:** 1 Faculty of Technology Novi Sad, University of Novi Sad, Bulevar cara Lazara 1, 21000 Novi Sad, Serbia; lj.spasojevic@tf.uns.ac.rs (L.S.); ljiljana04@tf.uns.ac.rs (L.P.); jelenamilinkovic@uns.ac.rs (J.M.B.); jadranka@uns.ac.rs (J.F.); lidijap@uns.ac.rs (L.P.); jaroslav.katona@uns.ac.rs (J.K.); 2 Mining and Metallurgical Institute, Satbayev University, Satbayev str. 22a, 050013 Almaty, Kazakhstan; a_sharipova85@mail.ru; 3 Petroleum Engineering Institute “One Belt, One Road”, Kazakh–British Technical University, Tole bi str. 59, 050000 Almaty, Kazakhstan; tempus_aidarova@mail.ru (S.A.); a_babayev@mail.ru (A.B.); r.sarsembekova@kbtu.kz (R.S.)

**Keywords:** silk fibroin, poly(acrylic acid), polymer/polymer interaction, polyelectrolyte complex (PEC), complex coacervation

## Abstract

Silk fibroin (SF) is a protein with many outstanding properties (superior biocompatibility, mechanical strength, etc.) and is often used in many advanced applications (epidermal sensors, tissue engineering, etc.). The properties of SF-based biomaterials may additionally be tuned by SF interactions with other (bio)polymers. Being a weak amphoteric polyelectrolyte, SF may form polyelectrolyte complexes (PECs) with other polyelectrolytes of opposite charge, such as poly(acrylic acid) (PAA). PAA is a widely used, biocompatible, synthetic polyanion. Here, we investigate PEC formation between SF and PAA of two different molecular weights (MWs), low and high, using various techniques (turbidimetry, zeta potential measurements, capillary viscometry, and tensiometry). The colloidal properties of SF isolated from *Bombyx mori* and of PAAs (MW, overlap concentration, the influence of pH on zeta potential, adsorption at air/water interface) were determined to identify conditions for the SF-PAA electrostatic interaction. It was shown that SF-PAA PEC formation takes place at different SF:PAA ratios, at pH 3, for both high and low MW PAA. SF-PAA PEC’s properties (phase separation, charge, and surface activity) are influenced by the SF:PAA mass ratio and/or the MW of PAA. The findings on the interactions contribute to the future development of SP-PAA PEC-based films and bioadhesives with tailored properties.

## 1. Introduction

Silk is a natural fibrous protein that represents one of the most preferred and valued biomaterials from the period of its discovery until today. Silk is a biomaterial produced by various types of insects, but the greatest commercial importance is silk produced by silkworms *Bombyx mori* and spiders such as *Nephila clavipes* [1,2]. Silk fibers obtained from *Bombyx mori* silkworm cocoons usually contain 72–81% SF and 19–28% silk sericin and small amounts of ash, waxes, dyes, and other compounds. In *B. mori* silk, two parallel SF fibers are coated with a glue-like protein sericin. While SF provides mechanical strength, sericin enables the compactness of silk fibers and the entire cocoons [2,3]. In order to use silk as a biomaterial, sericin must be removed from the silk fibers by a process called degumming. There are many ways to extract the sericin, but one of the most commonly employed and examined degumming processes involves boiling cut cocoons in a sodium carbonate solution [3,4,5].

SF is composed of three fractions: heavy chain (H), light chain (L), and small P25 glycoprotein. The heavy chain is hydrophobic and rigid (~390 kg/mol), while the light chain is flexible and hydrophilic with a lower MW (~26 kg/mol). These two chains are linked by disulfide bonds and form an H-L complex, which is connected to the small glycoprotein P25 (~25 kg/mol) via hydrophobic interactions [6,7,8]. Due to its natural hierarchical structure and high degree of crystallinity, SF cannot be directly dissolved or separated in water, but it can be dissolved in some concentrated aqueous or aqueous/organic salt solutions [9]. Dissolving SF using an aqueous solution of lithium bromide is one of the most commonly used dissolution methods.

SF has unique properties such as mechanical stability, biocompatibility, biodegradability, and good handling characteristics. It can be fabricated into various materials such as hydrogels, films, sponges, scaffolds, mats, and micro- and nanoparticles [10,11]. SF is considered a very attractive biopolymer material for use in advanced technologies such as epidermal sensors [10,12], edible films and barrier coatings in the food industry [13,14], tissue engineering [5,15,16], drug carriers with controlled release [6,17], wound healing [1,11,18], natural emulsion and foam stabilizers [19,20], etc. Due to its favorable properties and potential clinical application, SF has been approved as a biomaterial by the FDA (Food and Drug Administration) since 1993 [8].

Although natural SF materials possess many outstanding properties, they rarely meet all the highly specific requirements necessary for a particular application. Different methods can be used to design new SF-based materials with improved functional and structural properties. Blending and cross-linking SF with other macromolecules are often used for this purpose [15].

Polymer/polymer interactions may occur when two polymers are found jointly in the mixture. Generally, several types of interactions occur between macromolecules, such as intermolecular hydrogen bonding, intermolecular covalent bonding, and electrostatic and hydrophobic interactions [21]. Complexes formed through electrostatic interactions between two oppositely charged polymer chains are called polyelectrolyte complexes (PECs) [22]. Kabanov and Zezin (1984) first showed that PEC formation is a fast process which takes place in less than 5 ms [23]. The initially formed complex undergoes subsequent intracomplex restructuration (the formation of new bonds and/or change in the conformation of polymer chains) to produce the ordered secondary complex. The second step proceeds within the order of an hour. The final, third step, involves the aggregation of the intercomplexes, mainly through hydrophobic interactions, leading to the formation of complex aggregates [24]. PEC formation is driven by electrostatic interactions and by the entropy gain attained through the release of counterions during complex formation between two oppositely charged polyelectrolytes [25]. PEC formation and stability depends on many factors, such as ionic strength, temperature, MW and the concentration of polyelectrolytes (PEs), the mixing ratio of polymers, the type of ionic groups, and the solution pH. The charge and charge density of PEs vary depending on the solution pH [22,26].

SF interactions with both natural and synthetic polymers have been reported. Malay et al. investigated the interaction of oppositely charged SF and hyaluronic acid and the formation of their complexes at different conditions [27,28]. Ghaeli et al. monitored the interactions between collagen and SF during the formulation of their stable and one-phase mixture [29]. Bahardwaj et al. studied the formation of an SF/chitosan polyelectrolyte complex for tissue engineering application, while the complexation of SF and tannic acid through hydrogen bonding for the preparation of wood adhesives was investigated by Kim et al. [30,31]. Wang et al. investigated the influence of SF/poly(lactic acid) interactions on the preparation of their biocomposites [32]. Gao et al. reported SF complex formation with the in-house synthetized PAA of medium MW when mixing dilute SF and PAA solutions, however, without pH adjustment [33].

PAA is a synthetic polymer made by polymerizing acrylic acid monomers. It is biocompatible, biodegradable, water-soluble, and a superabsorbent polymer [34]. PAA is also a non-toxic polymer with bioadhesive properties due to the large number of carboxylic groups [35]. PAA is a mucoadhesive material and has good adhesion properties to the mucosa cells, as well as the ability to adhere to skin, bone, and teeth [36]. Based on its properties, PAA has a wide range of applications, including adhesives, packaging, coatings, pharmacy, cosmetics, drug delivery systems, medicine, dentistry, etc. [34].

In this paper, electrostatic interactions between SF isolated from *B. mori* cocoons and commercially available PAA of low and high MWs were investigated. The colloidal properties of SF and PAA were first characterized to identify conditions for SF-PAA PEC formation. Furthermore, SF-PAA complex formation obtained at different interaction conditions was investigated. Different techniques, such as gel electrophoresis, turbidimetry, zeta potential measurements, capillary viscometry, and tensiometry were used for this purpose.

## 2. Materials and Methods

### 2.1. Materials

*Bombyx mori* silkworm cocoons were obtained from a farm cooperative in Wuhan, China. Sodium carbonate (Na_2_CO_3_, anhydrous, 99.5%) was purchased from Loba Chemie Pvt. Ltd. (Mumbai, India). Lithium bromide (LiBr, anhydrous) was purchased from Sigma-Aldrich Co. (St. Louis, MO, USA), and dialysis tubing cellulose membrane (molecular weight cut off 14 kg/mol) was purchased from Sigma-Aldrich, Co. (St. Louis, MO, USA). Hydrochloric acid (HCl, 37%) was purchased from ZORKA Pharma-HEMIJA d.o.o. (Šabac, Serbia), and sodium hydroxide micropearls (NaOH) were purchased from Lach-Ner, s.r.o. (Neratovice, Czech Republic). PAAs of high and low MWs were purchased from Sigma-Aldrich, Co. (St. Louis, USA). MW = 1033,000 and MW = 1800 were reported by the supplier for the high MW PAA and the low MW PAA, respectively. All other chemicals were of analytical grade, except as specified, and were used without further purification. Distilled water was used as the solvent in all experiments.

### 2.2. Preparation of SF Solution

*Bombyx mori* cocoons were cut into small pieces and boiled for 30 min in a 0.5% (*w*/*v*) sodium carbonate solution, then rinsed in distilled water heated to 40 °C with constant stirring for 30 min. This procedure was repeated three times in order to degum SF. The degummed SF was dried overnight in ambient conditions. The dry and degummed SF fibers were dissolved in a 9.3 M aqueous lithium bromide solution at 60 °C for 90 min. The resulting solution was dialyzed against distilled water using a dialysis tubing membrane for 3 days to remove the salt. Dialysis was performed at room temperature with constant stirring. After dialysis, the solution was centrifuged for 20 min at 5000 rpm and filtered through an 8.0 µm pore size filter to remove any remaining impurities and undissolved fibers. The final concentration of the aqueous SF solution was determined gravimetrically by measuring the dry matter content and calculated using Equation (1):

*ω*(%) = (*m*/*m*_0_) × 100 (1)

where *m* is the dry matter mass and *m*_0_ SF solution mass, averaged over at least three measurements. All SF solutions were stored at 4 °C before further use.

### 2.3. Preparation of PAA Solution

PAA aqueous solutions of different concentrations, namely 0.1%, 0.5%, and 1.0% (*w*/*v*), were prepared by dissolving PAA powder in distilled water with constant stirring one day before use. The pH of the solutions was adjusted using 0.1 M or 1.0 M HCl.

High MW PAA solution at a concentration of 0.5 g/100 mL and low MW PAA solution at a concentration of 10 g/100 mL in 2 M NaOH were prepared using the same method as in water and were used for determining the PAA viscosity average molar mass.

### 2.4. Preparation of SF-PAA Mixtures

SA-PAA mixtures were prepared at room temperature by adding PAA solution to SF solution in different proportions. The pH of both solutions was adjusted to 3 before mixing. PAAs of two different MWs (the high MW PAA and the low MW PAA) were used for SF-PAA complex preparation. SF/high MW PAA mixtures were prepared by adding the required volume of 0.1% (*w*/*v*) PAA solution to 0.1% (*w*/*v*) SF solution to obtain mixtures with SF:PAA mass ratios of 100:0, 98:2, 95:5, 92:8, 90:10, 85:15, 80:20, 70:30, 60:40, 50:50, 40:60, 30:70, 20:80, 15:85, 10:90, 8:92, 5:95, 2:98, and 0:100. SF/low MW PAA mixtures were prepared by adding the required volume of 1.0% (*w*/*v*) PAA solution to 1.0% (*w*/*v*) SF solution to obtain mixtures with SF:PAA mass ratios of 100:0, 99:1, 98:2, 97:3, 95:5, 92:8, 90:10, 85:15, 80:20, 70:30, 60:40, 50:50, 40:60, 30:70, 20:80, 10:90, 5:95, and 0:100. The obtained mixtures were stirred on a magnetic stirrer for 3 min and left overnight to allow for enough time for the formation of the equilibrium SF-PAA complex.

A 0.5 g/100 mL high MW PAA solution at pH 3 was mixed with a 0.5 g/100 mL SF solution at pH 3 to obtain SF-PAA mixtures with various SF:PAA mass ratios and a total polymer concentration of 0.5 g/100 mL for viscosity characterization. The mixtures were left for 24 h to equilibrate before further use.

### 2.5. Viscosity Measurements

Viscosity measurements were employed to determine the overlap concentration of PAAs, the viscosity average molar mass of PAA samples, and to investigate SF-PAA complex formation. Viscosity measurements were performed using a Cannon capillary viscometer 50 E599 (Cannon Instrument Company, State College, PA, USA) immersed in a thermostatic bath set to a measuring temperature. Before measurements, each sample was allowed to equilibrate for at least 15 min at the measuring temperature to ensure it reached the desired temperature. The flow times of polymer solutions of different concentration and solvents were measured. Each measurement was repeated at least five times, and the average values were reported. The measurement results were expressed as relative viscosity (*ƞ_rel_*), specific viscosity (*ƞ_sp_*), and reduced viscosity (*ƞ_red_*):

*η_rel_* = *t*/*t*_0_ (2)

*η_sp_* = *t* − *t*_0_/t_0_ = *η_rel_*− 1 (3)

*η_red_* = *η_sp_*/*c* (100 mL/g) (4)

where *t* is the flow time of the polymer solution, *t*_0_ the flow time of the solvent, and *c* is the concentration of the polymer solution.

The intrinsic viscosity [*ƞ*] of the PAA samples was obtained graphically, by extrapolating *lnη_rel_*/*c* and *η_sp_*/*c* to zero polymer concentration, according to Equation (5) [37]:

[*η*] ≡ *lim*_*c*→0_ (*lnη_rel_*/*c*) ≡ *lim*_*c*→0_
*η_sp_*/*c* (5)

The overlap concentration (c*) of PAA was estimated as c* = 1/[*η*].

The viscosity average molar masses (Mvs) of PAA samples were calculated from the Mark–Houwink equation:

[*η*] = *K* × *Mv^a^* (6)

where *K* and *a* are constants characteristic for a particular solvent/polymer system at a given temperature. *K* = 42.2 × 10^−5^ 100 mL/g and *a* = 0.64 at 25 ± 0.1 °C as well as *K* = 53.9 × 10^−4^ 100 mL/g and *a* = 0.43 at 30 ± 0.1 °C were used for the low MW PAA and the high MW PAA Mv determination in 2 M NaOH aqueous solution, respectively [38,39].

Viscosimetric measurements of SF-PAA mixtures with different SF:PAA mass ratios, SF solutions of different concentrations, and PAA solutions of different concentrations were performed at 20 ± 0.1 °C and pH 3 to investigate SF-PAA complex formation. Viscosimetric measurements of supernatants were carried out in SF-PAA mixtures where sediment formation occurred.

### 2.6. Turbidity Measurements

Turbidity measurements were performed by using the UV-VIS HALO DB-20S Spectrophotometer (Dynamica Scientific Ltd., Livingston, UK). Transmittance at 500 nm in a 1 cm length cell in reference to distilled water was measured at room temperature. The turbidity of samples was calculated using Equation (7) [40]:

turbidity = 100 − transmittance (%)(7)

The turbidity of 1.0% (*w*/*v*) SF aqueous solutions at different pH values and the turbidity of SF-PAA mixtures at different SF:PAA mass ratios were determined. The total polymer concentration in SF-PAA mixtures was 0.1% (*w*/*v*) and 1.0% (*w*/*v*) for the high MW and low MW PAA mixtures, respectively. The turbidity of the supernatant was determined in all samples where sediment formation occurred.

All measurements were carried out in triplicate, and average values were calculated.

### 2.7. Zeta Potential Measurements

The zeta potential (ζ) of SF, PAA, and SF-PAA mixtures was measured using dynamic light scattering with the Zetasizer Nano ZS instrument (Malvern Instruments, Malvern, UK) at 25 °C. A folded capillary cell (DTS 1060) was used for zeta potential measurements.

The zeta potential of 0.1% (*w*/*v*) SF and PAA solutions at different pH (2.5–7.0) was determined. The pH of the samples was adjusted using 1.0 M or 0.1 M HCl.

The zeta potential of SF-PAA mixtures with total polymer concentration of 0.1% (*w*/*v*) and 1.0% (*w*/*v*), corresponding to the high and low MW PAA, respectively, was measured. These mixtures, prepared with different SF:PAA mass ratios, were characterized 24 h after preparation. The zeta potential of the supernatant was measured in mixtures where sediment formation occurred.

All measurements were performed in triplicate, and average values were reported.

### 2.8. Potentiometric Titration

The pI of SF was determined using potentiometric titration, where the pH of a 0.1% (*w*/*v*) SF solution was recorded during titration with 0.01 M HCl titrant at room temperature. The pH measurements were performed using 827 pH lab pH-meter (Metrohm, Herisau, Switzerland). The pI of SF was derived from the obtained titration curve based on the titration equivalent point. The equivalent point was determined from the obtained dependence of ∆pH/∆V on HCl volume.

### 2.9. Sodium Dodecyl Sulfate–Polyacrylamide Gel Electrophoresis (SDS-PAGE)

The MW of SF was determined by sodium dodecyl sulfate–polyacrylamide gel electrophoresis, described by the method of Laemmli [41] with certain corrections. Polyacrylamide gel electrophoresis (PAGE) in the presence of the anionic detergent, sodium dodecyl sulfate (SDS), has proven to be a useful method for protein separation and for the determination of their molecular weights. The gel system consists of a 4% (*w*/*v*) acrylamide stacking gel and a 12% (*w*/*v*) acrylamide separation gel. Samples were prepared by adding 0.5 mL of 2% (*w*/*v*) SF solution in 0.5 mL of Tris/Gly buffer (pH 6.8) which contains 20 g/L SDS and 50 g/L 2-mercaptoethanol. After the preparation of gels and samples, the apparatus for electrophoresis (Multi Drive XL, Pharmacia, Stockholm, Sweden) was put into operation at 50 mA, at 25 °C, as long as the dyed tracker reached the bottom of each gel. When electrophoresis was finished, the gels were stained with 0.2% (*w*/*v*) Coomassie brilliant blue R-250 in 10% (*v*/*v*) acetic acid. The gels were distained for 48 h with 10% (*v*/*v*) acetic acid which contains 40% (*v/v*) methanol.

### 2.10. Tensiometry

The surface tension measurements of 0.05% (*w*/*v*) PAA solution, SF solutions (0.01–2% (*w*/*v*)), and SF-PAA mixtures (0.05% (*w*/*v*) PAA and 0.01–2.00% (*w*/*v*) SF) were carried out by a KSV Sigma 703D tensiometer at 25 °C. The Du Noüy ring method was employed. Prior to the measurements, the air/liquid interface was disturbed by vigorous mixing, the ring was then immersed into the liquid (below the interface), and then the interface was left to equilibrate for 30 min. The reported values of the surface tension are average values of at least three measurements.

## 3. Results and Discussions

### 3.1. Colloidal Properties of SF and PAA

The colloidal properties of polymers (e.g., MW, charge, and pI) influence their behavior in solutions, as well as their interaction with other polymers when found jointly in mixtures. The MW, the influence of pH on zeta potential, and the pI of SF and PAA were determined.

#### 3.1.1. Determination of Molecular Weights

**Figure 1:**
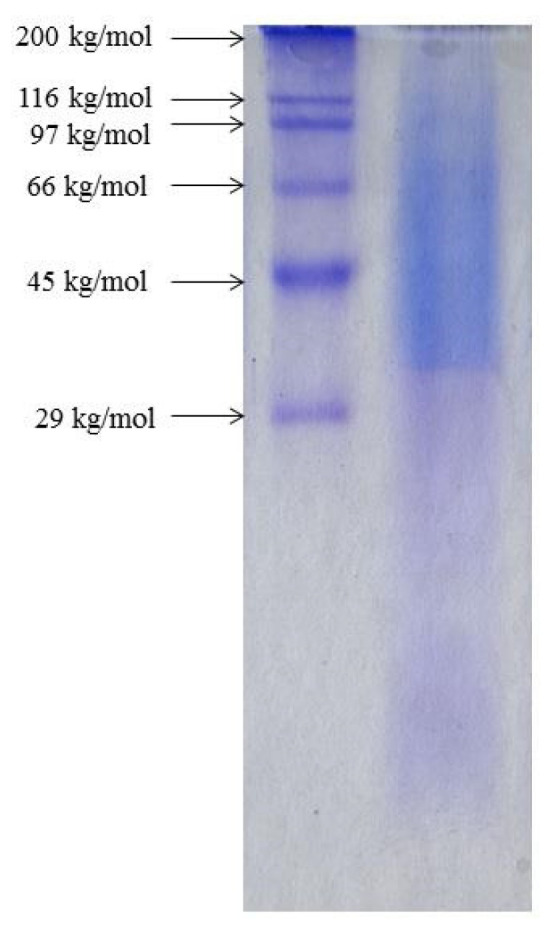
Sodium dodecyl sulfate–polyacrylamide gel electrophoresis (SDS-PAGE) for standard protein markers (lane 1) and for 2% (*w*/*v*) SF solution (line 2).

The MW of SF was determined by SDS-PAGE electrophoresis (Figure 1). It can be seen from Figure 1 that the molar mass of the isolated SF ranged from ≈30 kg/mol to ≈116 kg/mol, which is significantly lower than that found in native SF [6,7,8].

This suggests that the employed isolation procedure, which includes degumming in sodium carbonate solution, results in the cleavage of native SF chains and bonds between the SF heavy and light chains which eventually leads to a decrease in the MW of SF [42]. The native SF is therefore degraded during the isolation procedure, and the isolated SF represents a mixture of polypeptides of various MWs.

**Figure 2:**
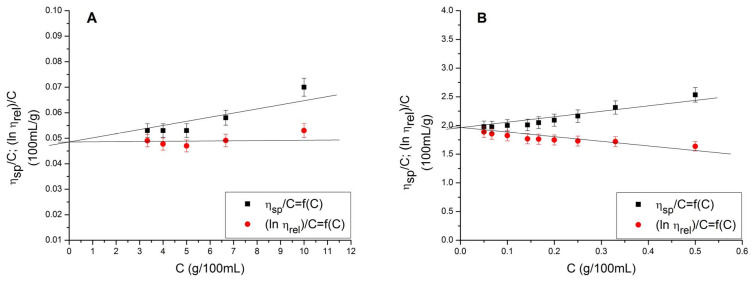
The double extrapolation method determination of intrinsic viscosity for (**A**) the low MW PAA and (**B**) the high MW PAA, both in 2 M NaOH solution.

Capillary viscometry of both low MW PAA and high MW PAA was carried out to determine the intrinsic viscosity of PAAs and their Mvs. The intrinsic viscosity was determined using the double extrapolation method, where experimentally obtained *η_sp_*/*c* and *lnη_rel_*/*c* values for PAA solutions of different concentrations were extrapolated to zero concentration, as illustrated in Figure 2A,B.

The intrinsic viscosity determined was 0.0485 100 mL/g (Figure 2A) for the low MW PAA and 1.965 100 mL/g (Figure 2B) for the high MW PAA. This gives c* = 20.62 g/100 mL and c* = 0.51 g/100 mL for the low and high MW PAA, respectively. PAA solutions with a concentration lower than c* are diluted, meaning that no interaction between individual PAA chains occurs [43].

The Mv of PAA samples was calculated from the experimentally obtained intrinsic viscosities using the Mark–Houwink equation (Equation (6)). It was determined that Mv = 1598 g/mol for the low MW PAA and Mv = 907,012 g/mol for the high MW PAA.

#### 3.1.2. Determination of Isoelectric Point

**Figure 3:**
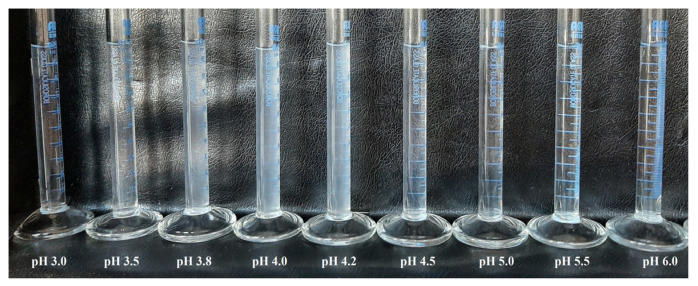
Photograph of 1.0% (*w*/*v*) SF solutions at different pHs.

The solution properties of weak polyelectrolytes such as SF and PAA are influenced by pH. Figure 3 shows photograph of SF solutions at different pH values (3–6). It can be seen from Figure 3 that the visual appearance of the solutions is influenced by the solution pH. The solutions with low and high pH values are clear and transparent, while the solutions with intermediate pH values appear turbid.

**Figure 4:**
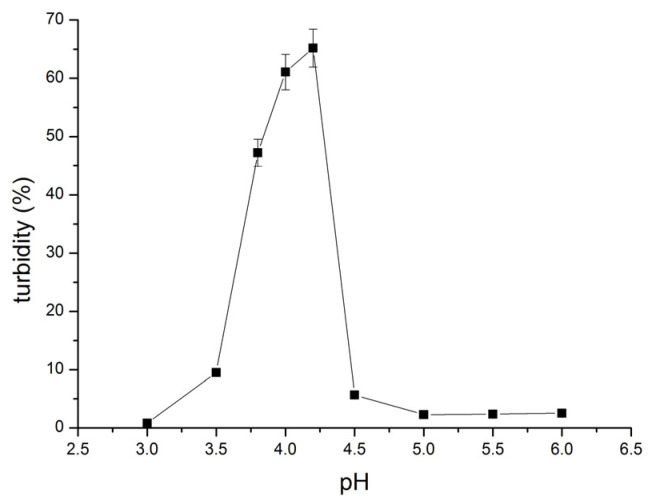
Influence of pH on turbidity of 1.0% (*w*/*v*) SF solutions.

The turbidity of SF solutions with different pHs was measured, and the influence of the solution pH on turbidity is shown in Figure 4.

From Figure 4, it can be seen that the turbidity of SF solutions increases with an increase in pH, reaching maximum turbidity at pH 4.2. A further increase in the solution pH brings about a sharp decrease in turbidity to reach a constant, low turbidity value for pH > 5. Unlike polymer solutions in good solvents, which do not scatter light, colloidal dispersions possess this ability, and as a result of light scattering, turbidity occurs. The increased turbidity of SF solutions indicates the phase separation of the SF solution, where the aggregation of SF fibrils takes place due to the low charge of SF molecules and the subsequently decreased solubility of SF. Namely, being a weak, amphoteric polyelectrolyte, the charge of SF molecules depends on the solution pH. At a low pH (lower than pI) SF is positively charged, facilitating good solubility and resulting in clear SF solutions. As the pH increases, the positive charge on SF molecules decreases while the negative charge increases, effectively reducing the net charge of SF. This reduction in charge decreases the solubility of SF and leads to an increase in the turbidity of the solutions. At pI, the amount of positive and negative charge in the SF molecule is balanced and SF becomes effectively uncharged and least soluble. This balance brings about phase separation, resulting in the maximum turbidity of the solution. Figure 3 shows the maximum in the turbidity of the SF solution and thereby pI = 4.2. As the pH increases above the pI, SF molecules become more negatively charged. This increased negative charge enhances the solubility of SF, thereby reducing the turbidity of the SF solution.

**Figure 5:**
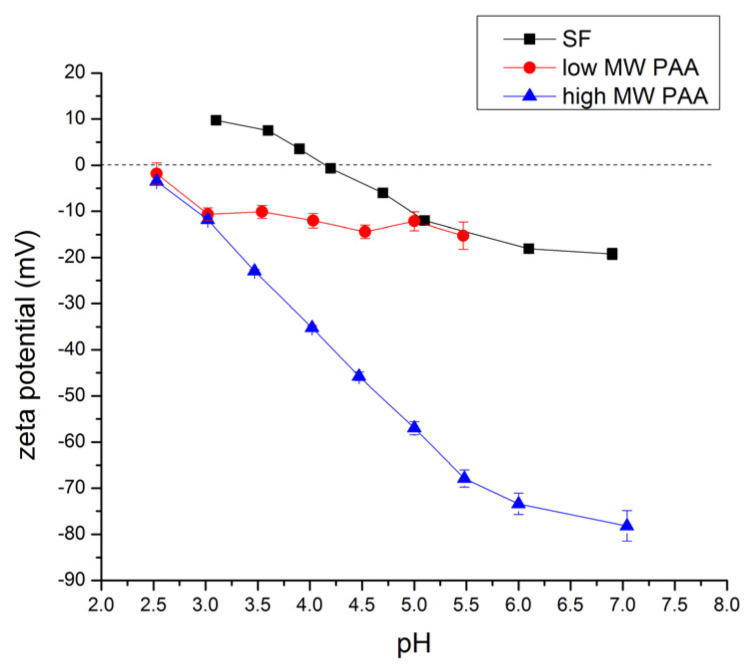
Influence of pH value on zeta potential of 0.1% (*w*/*v*) SF and PAA aqueous solution.

Such an influence of the solution pH on the charge of SF molecules was confirmed by zeta potential measurements (Figure 5). Freshly prepared SF solutions after dialysis typically have a pH ranging from 7 to 8, depending on the concentration of the SF solution. As shown in Figure 5, at this pH, SF is negatively charged and behaves as a polyanion. A decrease in the SF solution pH brings about a gradual increase in the SF zeta potential from −19.20 mV at pH 6.9 to 0 mV at pH 4.2. With a further decrease in pH, SF becomes positively charged with a zeta potential of 9.73 mV observed at pH 3.1. The zero value of the zeta potential was obtained at pI = 4.2. This is in agreement with the literature data, where reported values of SF’s pI range from 3.6 to 5.2 [9,19,44].

Figure 5 also shows the influence of the pH on the zeta potential of both the low MW and the high MW PAA. In both cases, the low MW and the high MW PAA solutions exhibited a negative zeta potential across the entire investigated pH range, typical for polyanions. At the lowest pH studied (pH 2.53), the zeta potential was close to zero due to the hindered dissociation of carboxylic groups. An increase in pH brings about the increased dissociation of carboxylic groups and thereby an increase in the negative charge of PAA. The increase in negative charge is significantly more pronounced for the high MW PAA compared to the low MW PAA, as shown in Figure 5.

**Figure 6:**
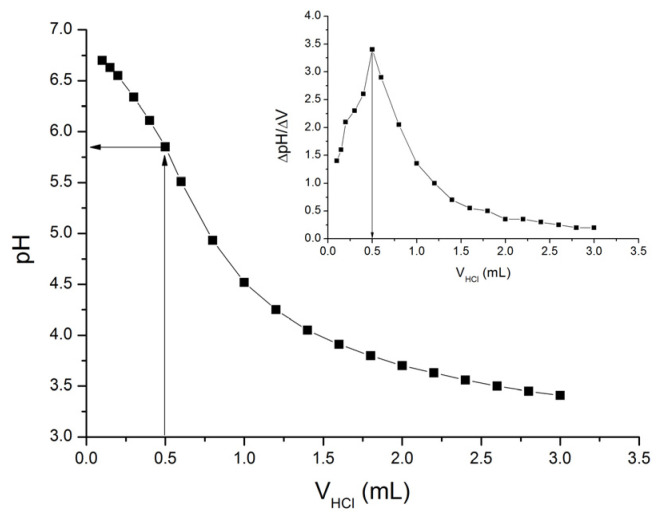
Titration curve of 0.1% (*w*/*v*) SF solution titrated with 0.01 M HCl, obtained at room temperature.

Potentiometric titration with a suitable acid or base is another convenient method used for pI determination. The influence of HCl volume added to the SF solution on the pH of the SF solution is shown in Figure 6. The equivalent titration point was determined from the first derivative of the obtained dependence and was found to be 0.5 mL of HCl (inset to Figure 6). The obtained data for the equivalent titration point suggests that the pI of SF ranges from 5.50 to 6.25. The pI obtained by potentiometric titration is somewhat higher than the pI obtained by turbidity measurements (Figure 4) and by zeta potential measurements (Figure 5).

### 3.2. Investigation of SF-PAA Complexes Formation

The investigation of the zeta potential (Figure 5) showed that SF was in the protonated form and positively charged below pH 4.2. Conversely, above pH 2.5, both low and high MW PAAs were in the deprotonated forms, and therefore negatively charged. This suggests that the electrostatic interaction between SF and PAAs and PEC formation is expected to take place when both polymers are found in a solution at a pH which is higher than 2.5 and lower than 4.2. Therefore, mixtures of SF with both low MW PAA and high MW PAA were prepared at pH 3 and at different SF:PAA mass ratios.

#### 3.2.1. Turbidity Measurements

**Figure 7:**
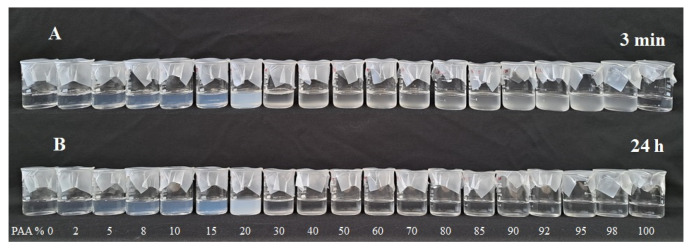
Photograph of 1.0% (*w*/*v*) SF/low MW PAA mixtures at different SF:PAA mass ratios (shown as PAA%), (**A**) 3 min after preparation and (**B**) 24 h after preparation.

Photographs of 1.0% (*w*/*v*) SF/low MW PAA mixtures at pH 3 and at different SF:PAA mass ratios, taken 3 min and 24 h after preparation, are shown in Figure 7.

It can be seen from Figure 7 that the 100% SF solution (0% PAA) and the 100% low MW PAA solution (0% SF) remained clear at both 3 min and 24 h after preparation. However, SF-PAA mixtures at all investigated SF:PAA mass ratios immediately became turbid after preparation, indicating the formation of SF/low MW PAA PECs. The complex formation is attributed to electrostatic interactions of positively charged SF and negatively charged PAA at pH 3 (Figure 5). PEC formation is also driven by the entropy gain attained through the release of counterions during complex formation between two oppositely charged polyelectrolytes [25]. Moreover, it was reported previously that hydrogen bonding between PAA’s carboxylic groups and SF’s amide groups can also contribute to SF-PAA complex formation [33].

**Figure 8:**
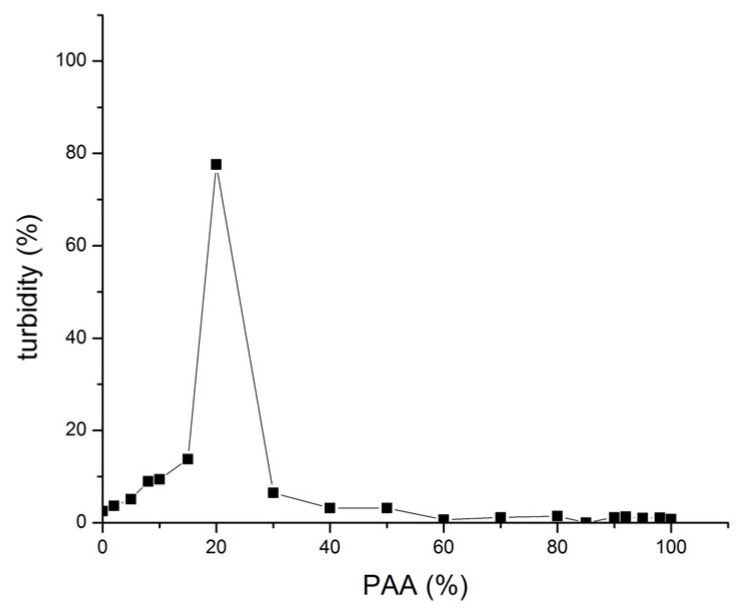
Turbidity of 1.0% (*w*/*v*) SF/low MW PAA mixtures at different SF:PAA mass ratios (shown as PAA%), at pH 3, 24 h after mixing.

Figure 8 shows that SF-PAA mixtures containing 2% to 20% of PAA remained turbid after 24 h. SF-PAA mixtures containing 30% to 80% PAA cleared overnight, with a significant decrease in turbidity observed 24 h after preparation (Figure 8). The decrease in turbidity was due to PEC precipitation and sediment formation, which were visually observed in the mixtures. The highest yield of precipitate occurred at about 30–40% PAA and could be easily resuspended by stirring with a glass rod. The precipitate formed at 50–70% PAA was sticky and could not be resuspended by stirring, while the precipitate at 80% was barely noticeable on the walls of the beaker. No sediment formation was observed for SF/low MW PAA mixtures containing >80% PAA.

**Figure 9:**
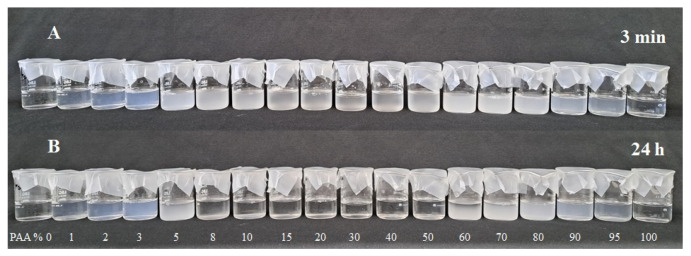
Photograph of 0.1% (*w*/*v*) SF/high MW PAA mixtures at different SF:PAA mass ratios (shown as PAA%), (**A**) 3 min after preparation and (**B**) 24 h after preparation.

SF/high MW PAA PEC formation was also investigated. Figure 9 shows 0.1% (*w*/*v*) SF/high MW PAA mixtures at pH 3 and at different SF:PAA mass ratios, 3 min and 24 h after preparation.

It can be seen from Figure 9 that SF/high MW PAA mixtures for all SF:PAA mass ratios (except for 100% PAA and 100% SF) were turbid 3 min after preparation, suggesting SF/high MW PAA PEC formation. Mixtures with 8% to 50% of PAA cleared overnight due to PEC sediment formation. The precipitate yield was the highest at about 8–10% PAA and could be easily resuspended by stirring with a glass rod. The precipitate formed at 15–20% PAA was sticky and could not be resuspended by stirring, while the precipitate at 30–50% was barely noticeable at the bottom and on the walls of the beaker.

**Figure 10:**
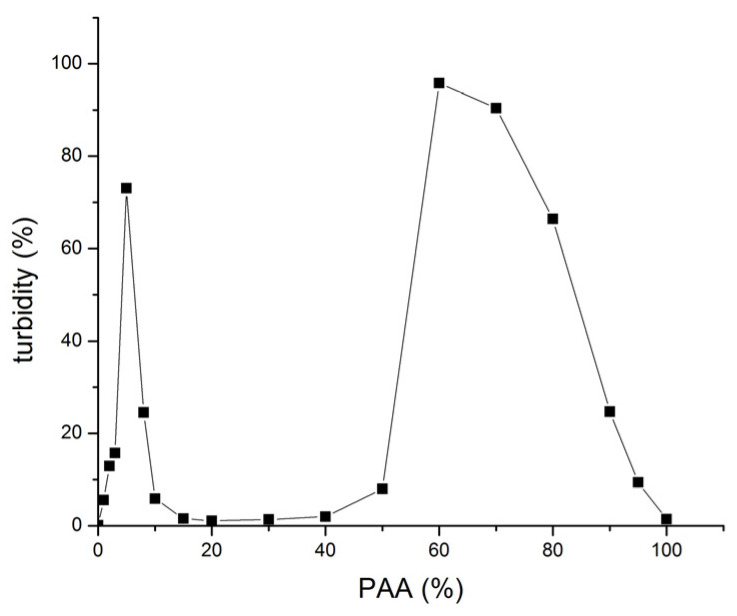
Turbidity of 0.1% (*w/v*) SF/high MW PAA mixtures at different SF:PAA mass ratios (shown as PAA%), at pH 3, 24 h after mixing.

The turbidity of 0.1% (*w*/*v*) SF/high MW PAA mixtures at different SF:PAA mass ratios (shown as PAA%), at pH 3, 24 h after mixing is shown in Figure 10.

Turbidity increases with an increase in the PAA% up to 5% of PAA. This increase is attributed to the formation of more PECs as more PAA is available for interacting with SF and/or a decrease in PEC solubility likely due to a decrease in the net positive charge of the PEC. The turbidity of SF-PAA mixtures with 10% to 50% of PAA drops to close to zero values due to the sedimentation of more or less electroneutral PEC formation. However, a further increase in the PAA% in the mixture gradually increases the net negative charge of the PEC, resulting in the redispersion of the complex and a subsequent sharp increase in the turbidity of the SF-PAA mixture with 60% of PAA. A further increase in the PAA% brings about a decrease in the turbidity of the mixtures (Figure 10). This decrease in turbidity may be attributed to an increase in the complex solubility due to an increase in the net negative charge of the complex and/or to a decrease in the amount of the complex formed, as less and less SF is present in the system when the PAA% approaches 100%.

#### 3.2.2. Zeta Potential Measurements

**Figure 11:**
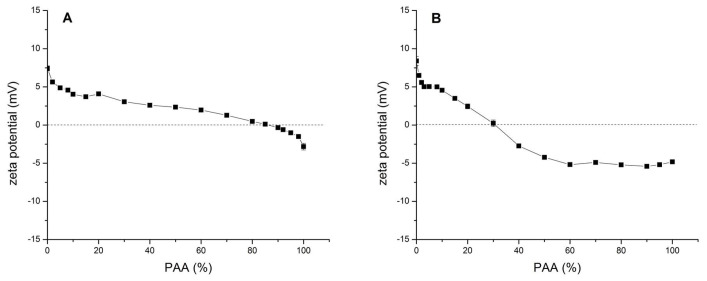
Zeta potential of SF-PAA mixtures at different SF:PAA mass ratios (shown as PAA%), at pH 3, 24 h after mixing: (**A**) 1.0% (*w*/*v*) SF/low MW PAA mixtures; (**B**) 0.1% (*w*/*v*) SF/high MW PAA mixtures.

The influence of the PAA content in SF-PAA mixtures on the zeta potential of the SF PEC with both the low MW and the high MW PAA is shown in Figure 11A and Figure 11B, respectively.

A net positive zeta potential of SF/low MW PAA complexes was observed in all SF-PAA mixtures where the PAA% was less than 85 (Figure 11A). The net positive zeta potential gradually decreases with an increase in the PAA% until it reaches negative values at a PAA% higher than 90. It should be observed, though, that the measured zeta potential values, both positive and negative, are very close to zero. This indicates that subtle changes in the system determine the phase behavior of the PEC.

SF/high MW PAA PEC is positively charged for all SF-PAA mixtures with less than 30% PAA, when the zeta potential reaches zero (Figure 11B). When the PAA% in the mixture is higher than 30, the PEC becomes negatively charged. The net negative charge of the complex decreases up to 60% of PAA in the mixture. Further increases in the PAA% do not result in any additional changes in the zeta potential of the SF/high MW PAA PEC. This suggests that a decrease in turbidity observed for SF/high MW PAA mixtures at a PAA% higher than 60% (Figure 10) is most likely due to a decrease in the amount of complex formed, rather than a change in the solubility of the complex.

A comparison of Figure 11A,B shows that SF-PAA complex formation is influenced by the MW of PAA. The addition of the PAA solution to the SF solution at pH 3 leads to PEC formation, which is positively charged for the mixtures with a low PAA%, for both low and high MW PAA. An increase in the PAA% in the mixture results in a decrease in the net positive charge of the PEC. However, the decrease is more rapid for the high MW PAA, and the complex becomes negatively charged at a lower PAA% compared to the low MW PAA.

#### 3.2.3. Capillary Viscometry of SF/High MW PAA Mixtures

**Figure 12:**
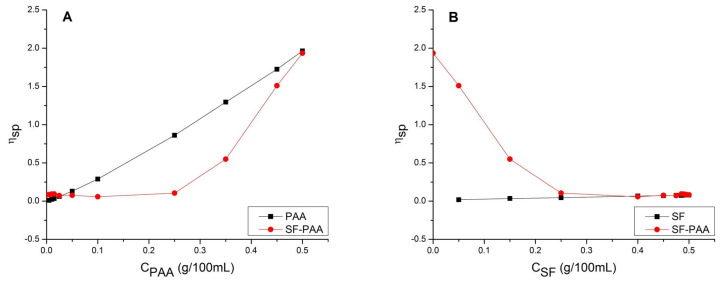
(**A**) Influence of PAA concentration on specific viscosity of PAA solution and SF-PAA mixture with different SF:PAA mass ratios (100% PAA in mixture is at 0.5 g/100 mL PAA), both at pH 3 and 24 h after preparation. (**B**) Influence of SF concentration on specific viscosity of SF solution and SF-PAA mixture with different SF:PAA mass ratios (100% SF in mixture is at 0.5 g/100 mL SF), both at pH 3 and 24 h after preparation.

The investigation of PEC formation in 0.5 g/100 mL SF/high MW PAA mixtures of different SF:PAA mass ratios at pH 3 was conducted using capillary viscometry 24 h after the mixture preparation. Figure 12A shows the influence of the PAA concentration on the specific viscosity of the PAA solution. It also shows the specific viscosity of SF-PAA mixtures with different SF:PAA mass ratios (the mixture with 0.5 g/100 mL PAA is considered 100% PAA, while the mixture with 0 g/100 mL PAA is considered 100% SF).

It can be seen from Figure 12A that the specific viscosity of the PAA solution constantly increases with an increase in PAA concentration. On the other hand, the specific viscosity of the SF-PAA mixture does not change with an increase in the PAA concentration and remains almost constant until the PAA concentration reaches 0.25 g/100 mL (i.e., from 0% to 50% PAA in the mixture). For most of the concentration range, the specific viscosity of the SF-PAA mixture is significantly lower than that of the PAA solutions. Since at this concentration range, SF-PAA complex formation already takes place (Figure 9), the difference in specific viscosity suggests that the SF-PAA PEC’s contribution to the specific viscosity of the SF-PAA mixture is significantly lower than the contribution of freely dissolved PAA molecules. Moreover, the constant specific viscosity of SF-PAA mixtures in the 0.00–0.25 g/100 mL PAA concentration range suggests that all of the PAA present in the mixture is entirely used for SF-PAA complex formation and that there are almost no uncomplexed, freely dissolved PAA molecules. A further increase in PAA concentration, above 0.25 g/100 mL, results in a sharp increase in the specific viscosity of SF-PAA mixtures (Figure 12A). At the same time, the difference in the specific viscosity of the PAA solution and SF-PAA mixture, for the same PAA concentration, decreases. The sharp increase and the reduced difference in specific viscosity indicate the presence of a surplus of uncomplexed, freely dissolved PAA in the SF-PAA mixture. This is attributed to the fact that in SF-PAA mixtures with a high PAA%, there is not enough SF to complex all of the dissolved PAA, and therefore some of the PAA remains uncomplexed.

Figure 12B shows the influence of SF concentration on the specific viscosity of the SF solution. It also shows the specific viscosity of SF-PAA mixtures with different SF:PAA mass ratios (where the mixture with 0.5 g/100 mL SF represents 100% SF, and the mixture with 0 g/100 mL SF represents 100% PAA). It can be seen from Figure 12B that the specific viscosity of the SF oligomers solution is low and only slightly increases with an increase in SF concentration. Therefore, the contribution of freely dissolved SF to the specific viscosity of SF-PAA mixtures can be neglected for most of the SF-PAA mixture compositions. The contribution is observed only for the lowest PAA concentrations (i.e., the highest SF concentrations) in the mixtures (Figure 12A) when there is not enough PAA to complex all of the dissolved SF present in the mixture, thereby leaving most of the SF uncomplexed and freely dissolved. The dissolved SF makes the specific viscosity of SF-PAA mixtures with *C_PAA_* < 0.025 g/100 mL slightly higher than the specific viscosity of the PAA solution (Figure 12A). The viscosimetric study showed that the viscous behavior of SF-PAA mixtures is predominantly determined by the colloidal state of PAA molecules.

#### 3.2.4. Tensiometric Investigation of SF/High MW PAA Mixtures

**Figure 13:**
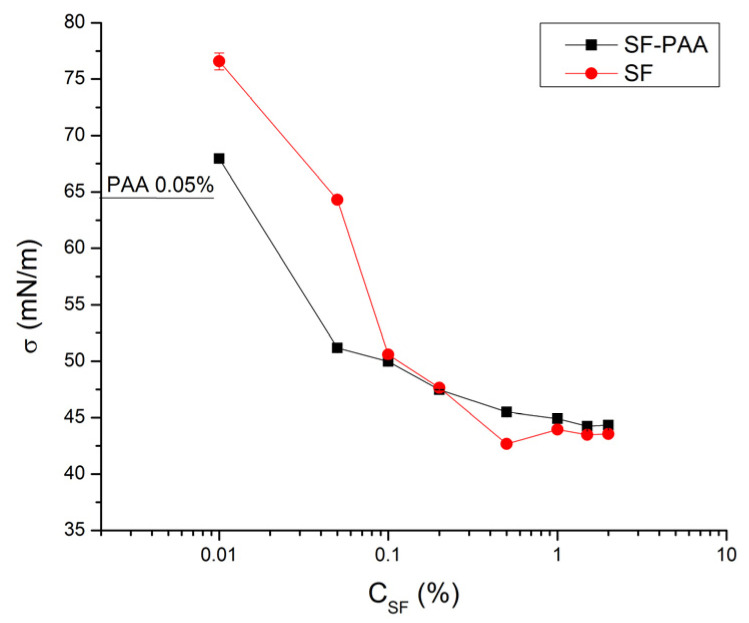
Influence of SF concentration on surface tension of SF solutions and surface tension of SF/high MW PAA mixtures (0.05% (*w*/*v*) PAA and 0.01–2.00% (*w*/*v*) SF), at pH 3.

The influence of SF concentration on the surface tension of SF solutions (0.01–2.00% (*w*/*v*)) and the surface tension of SF/high MW PAA mixtures (0.05% (*w*/*v*) PAA and 0.01–2.00% (*w*/*v*) SF), both at pH 3, is shown in Figure 13. Figure 13 shows that the surface tension of SF solutions decreases with an increase in SF concentration. The decrease is faster at lower SF concentrations (<0.1%); it slows down at the intermediate SF concentrations (0.1–1.0%) and reaches a plateau value for SF concentrations >1%. The obtained dependence is typical for surface active molecules and shows that the decrease in surface tension is due to the adsorption of SF at the air/water interface. The obtained SF surface tension isotherm does not have a sharp break point typical for the critical micelle concentration (CMC), which suggests that the onset of micelle-like SF aggregate formation is not that well defined as is the case in typical surfactant solutions, most likely due to the polydispersity of SF molecules [45]. As suggested by the isotherm, the onset of SF micelle-like aggregate formation most likely takes place in the 0.1–0.5% SF range.

The surface tension of 0.05% (*w*/*v*) PAA at pH 3 was c.a. 65 mN/m (Figure 13), which suggests that some of the PAA molecules adsorb at the air/water interface. PAA, a highly charged polyanion, is typically well soluble in water and does not adsorb at the air/water interface at a solution pH > 4. However, at pH 3, the dissociation of carboxylic groups is limited, which leads to a decrease in the PAA charge and consequently makes PAA macromolecules surface-active [46].

The influence of SF concentration on the surface tension of SF/high MW PAA mixtures with 0.05% (*w*/*v*) PAA is shown in Figure 13. The figure shows that the surface tension of the mixture sharply decreases with an increase in SF concentration when the SF concentration was less than 0.05% (*w*/*v*). In this region, the surface tension of the mixture is significantly lower than the surface tension of corresponding SF solutions, which is attributed to the formation of surface-active SF-PAA electrostatic complexes which form at the air/water interface [47]. A further increase in SF concentration (above 0.05% (*w*/*v*) SF) brings about a further decrease in the surface tension of SF-PAA mixtures, but this time the decrease is significantly slower. The break point of the SF-PAA mixture surface tension isotherm observed at 0.05% (*w*/*v*) SF corresponds to the critical aggregation concentration (CAC), i.e., to the formation of micelle-like aggregates of SF bound to PAA [48]. The CAC was found to be lower than the CMC of pure SF solutions. The onset of SF-PAA complex formation in the bulk (i.e., CAC) was also indicated by the turbidity change in the mixtures, where the SF-PAA mixture at 0.01% (*w*/*v*) SF concentration was clear, while the mixtures with SF concentrations higher than 0.01% (*w*/*v*) were turbid or clear with sediment coacervate phase (not shown). The surface tension of SF-PAA mixtures with SF concentrations >0.05% (*w*/*v*) becomes higher than the surface tension of the corresponding SF solutions and eventually, for the highest SF concentrations studied, approaches the surface tension of the corresponding SF solutions (Figure 13). The surface tension of the mixture becomes equal to the surface tension of SF when the air/water interface in the mixture is entirely covered by SF molecules while all of the PAA is complexed with SF in the bulk, which is expected to take place for the investigated SF-PAA mixtures at SF concentrations higher than 2.0%.

## 4. Conclusions

The investigation into the colloidal properties of SF and PAA showed that the two polymers can form PECs through electrostatic interaction when the pH of the SF-PAA mixture is higher than 2.5 and lower than 4.2. In this pH range, SF is in its protonated form and positively charged, while PAA is in its deprotonated form and negatively charged. SF-PAA complex formation occurred at all investigated SF:PAA mass ratios (except for 100:0 and 0:100) when the two polymers were mixed at pH 3 and the total polymer concentration at least 0.1% (*w*/*v*).

The SF complex with the low MW PAA is slightly positively charged for SF:PAA mass ratios ranging from 98:2 up to 20:80 and becomes slightly negatively charged from 10:90 up to 2:98 mass ratios. The positively charged complex remains dispersed even 24 h after preparation when the PAA% in the SF-PAA mixture is 20 or less. The precipitated complex forms sediment when the PAA% in the mixture ranges from 30 to 80.

The SF complex with the high MW PAA is slightly positively charged for SF:PAA mass ratios ranging from 99:1 up to 70:30 and becomes slightly negatively charged from 60:40 up to 5:95 mass ratios. The positively charged complex remains dispersed even 24 h after preparation when the PAA% in the SF-PAA mixture is 5% or less. The precipitated complex forms sediment when the PAA% in the mixture ranges from 8% to 50%. The negatively charged SF-PAA complex remains dispersed in the mixture for at least 24 h when the PAA% ranges from 60 to 95%.

A viscosimetric study on SF/high MW PAA mixtures suggests that practically all of the PAA is bound to SF when the PAA% in the SF-PAA mixture is less than 50%.

The study on SF-PAA electrostatic interactions enables the formation of SF-PAA PECs of tailored properties, which can readily be employed in future studies on designing SF-PAA PEC-based biomaterials, such as biopolymer films and bioadhesives, with advanced properties.

## Data Availability

The raw data supporting the conclusions of this article will be made available by the authors on request.

## References

[1] Gholipourmalekabadi M., Sapru S., Samadikuchaksaraei A., Reis R.L., Kaplan D.L., Kundu S.C. (2020). Silk fibroin for skin injury repair: Where do things stand?. Adv. Drug Deliv. Rev..

[2] Patel M., Dubey D.K., Singh S.P. (2020). Phenomenological models of *Bombyx mori* silk fibroin and their mechanical behavior using molecular dynamics simulations. Mater. Sci. Eng. C.

[3] Partlow B.P., Tabatabai A.P., Leisk G.G., Cebe P., Blair D.L., Kaplan D.L. (2016). Silk Fibroin Degradation Related to Rheological and Mechanical Properties. Macromol. Biosci..

[4] Allardyce B.J., Rajkhowa R., Dilley R.J., Atlas M.D., Kaur J., Wang X. (2016). The impact of degumming conditions on the properties of silk films for biomedical applications. Text. Res. J..

[5] Bucciarelli A., Greco G., Corridori I., Pugno N.M., Motta A. (2021). A design of experiment rational optimization of the degumming process and its impact on the silk fibroin properties. ACS Biomater. Sci. Eng..

[6] Mottaghitalab F., Farokhi M., Shokrgozar M.A., Atyabi F., Hosseinkhani H. (2015). Silk fibroin nanoparticle as a novel drug delivery system. J. Control. Release.

[7] Nguyen T.P., Nguyen Q.V., Nguyen V.H., Le T.H., Huynh V.Q.N., Vo D.V.N., Trinh Q.T., Kim S.Y., Le Q.V. (2019). Silk Fibroin-Based Biomaterials for Biomedical Applications: A Review. Polymers.

[8] Qi Y., Wang H., Wei K., Yang Y., Zeng R.-Y., Kim I.S., Zhang K.-Q. (2017). A review of structure construction of silk fibroin biomaterials from single structures to multi-level structures. Int. J. Mol. Sci..

[9] Sashina E.S., Bochek A.M., Novoselov N.P., Kirichenko D.A. (2006). Structure and Solubility of Natural Silk Fibroin. Russ. J. Appl. Chem..

[10] Yao X., Zou S., Fan S., Niu Q., Zhang Y. (2022). Bioinspired silk fibroin materials: From silk building blocks extraction and reconstruction to advanced biomedical applications. Mater. Today Bio.

[11] Patil P.P., Reagan M.R., Bohara R.A. (2020). Silk fibroin and silk-based biomaterial derivatives for ideal wound dressings. Int. J. Biol. Macromol..

[12] Liu X., Liu J., Wang J., Wang T., Jiang Y., Hu J., Liu Z., Chen X., Yu J. (2020). Bioinspired, microstructured silk fibroin adhesives for flexible skin sensors. ACS Appl. Mater. Interfaces.

[13] Ruggeri E., Kim D., Cao Y., Fare S., De Nardo L., Marelli B. (2020). A multilayered edible coating to extend produce shelf life. ACS Sustain. Chem. Eng..

[14] Marelli B., Brenckle M.A., Kaplan D.L., Omenetto F.G. (2016). Silk Fibroin as Edible Coating for Perishable Food Preservation. Sci. Rep..

[15] Grabska-Zielinska S., Sionkowska A. (2021). How to improve physico-chemical properties of silk fibroin materials for biomedical applications?—Blending and cross-linking of silk fibroin—A review. Materials.

[16] Wani S.U.D., Gautama S.P., Qadrie Z.L., Gangadharappa H.V. (2020). Silk fibroin as a natural polymeric based bio-material for tissue engineering and drug delivery systems-A review. Int. J. Biol. Macromol..

[17] Pandey V., Haider T., Jain P., Gupta P.N., Soni V. (2020). Silk as a leading-edge biological macromolecule for improved drug delivery. J. Drug Deliv. Sci. Technol..

[18] Farokhi M., Mottaghitalab F., Fatahi Y., Khademhosseini A., Kaplan D.L. (2018). Overview of Silk Fibroin Use in Wound Dressings. Trends Biotechnol..

[19] Qiao X., Miller R., Schneck E., Sun K. (2020). Influence of pH on the surface and foaming properties of aqueous silk fibroin solutions. Soft Matter.

[20] Rao J.-J., Chen Z.-M., Chen B.-C. (2009). Modulation and Stabilization of Silk Fibroin-Coated Oil-in-Water Emulsions. Food Technol. Biotechnol..

[21] Shang S., Zhu L., Fan J. (2013). Intermolecular interactions between natural polysaccharides and silk fibroin protein. Carbohydr. Polym..

[22] Meka V.S., Sing M.K.G., Pichika M.R., Nali S.R., Kolapalli V.R.M., Kesharwani P. (2017). A comprehensive review on polyelectrolyte complexes. Drug Discov. Today.

[23] Kabanov V.A., Zezin A.B. (1984). Soluble interpolymeric complexes as a new class of synthetic polyelectrolites. Pure Appl. Chem..

[24] Kulkarni A.D., Vanjari Y.H., Sancheti K.H., Patel H.M., Belgamwar V.S., Surama S.J., Pardeshi C.V. (2016). Polyelectrolyte complexes: Mechanism, critical experimental aspects, and applications. Artif. Cells Nanomed. Biotechnol..

[25] KayitmazeR A.B., Seeman D., Minsky B.B., Dubin P.L., Xu Y. (2013). Protein-polyelectrolyte interactions. Soft Matter.

[26] Srinivas L., Ramana Murthy K.V. (2010). Preparation and evaluation of polyelectrolyte complex for oral controlled drug delivery. Asian J. Pharm..

[27] Malay Ö., Bayraktar O., Batigün A. (2007). Complex coacervation of silk fibroin and hyaluronic acid. Int. J. Biol. Macromol..

[28] Malay Ö., Batigün A., Bayraktar O. (2009). pH-and electro-responsive characteristics of silk fibroin-hyaluronic acid polyelectrolyte complex membranes. Int. J. Pharm..

[29] Ghaeli I., De Moraes M.A., Bepp M.M., Lewandowska K., Sionkowska A., Ferreira-da-Silva F., Ferraz M.P., Monteiro F.J. (2017). Phase Behaviour and Miscibility Studies of Collagen/Silk Fibroin Macromolecular System in Dilute Solutions and Solid State. Molecules.

[30] Bhardwaj N., Kundu S.C. (2011). Silk fibroin protein and chitosan polyelectrolyte complex porous scaffolds for tissue engineering applications. Carbohydr. Polym..

[31] Kim E., Jung J.-S., Yoon S.-G., Park W.H. (2023). Eco-friendly silk fibroin/tannic acid coacervates for humid and underwater wood adhesives. J. Colloid Interface Sci..

[32] Wang F., Wu H., Venkataraman V., Hu X. (2019). Silk fibroin-poly(lactic acid) biocomposites: Effect of protein-synthetic polymer interactions and miscibility on material properties and biological responses. Mater. Sci. Eng. C.

[33] Gao Q., Shao Z., Sun Y., Lin H., Zhou P., Yu T. (2000). Complex Formation of Silk Fibroin with Poly(acrylic acid). Polym. J..

[34] Arakaban H., Barani M., Akbarizadeh M.R., Pal Singh Chauhan N., Jadoun S., Dehghani Soltani M., Zarrintaj P. (2022). Polyacrylic acid Nanoplatforms: Antimicrobial, Tissue Engineering, and Cancer Theranostic Applications. Polymers.

[35] Pourmadadi M., Farokh A., Rahmani E., Mahdi Eshaghi M., Aslani A., Rahdar A., Ferreira L.F.R. (2023). Polyacrylic acid mediated targeted drug delivery nano-systems: A review. J. Drug Deliv. Sci. Technol..

[36] Ito T., Yamaguchi S., Soga D., Yoshimoto T., Koyama Y. (2022). Preparation of a Bioadhesive Poly(acrylic acid)/Polyvinyl pyrollidone Complex Gel and Its Clinical Effect on Dental Hemostasis. Gels.

[37] Xiong X.-P., Ke Q.-R., Zhu S.-Q. (2014). Introduction of a reliable method for determination of intrinsic viscosity for any polymer with high precision. Chin. J. Polym. Sci..

[38] Brandrup J., Immergut E.H., Grulke E.A., Abe A., Bloch D.R. (2005). Polymer Handbook.

[39] Sovilj V. (1996). Fizička Hemija Polimera.

[40] Petrović L.B., Milinković J.R., Fraj J.L., Bučko S.Đ., Katona J.M. (2016). An investigation of chitosan and sodium dodecyl sulfate interactions in acetic media. J. Serb. Chem. Soc..

[41] Laemmli U.K. (1970). Cleavage of structural proteins during the assembly of the head of bacteriophage T4. Nature.

[42] Guo C., Li C., Kaplan D.L. (2020). Enzymatic Degradation of *Bombyx mori* Silk Materials: A Review. Biomacromolecules.

[43] Tirrel M., Goddard D.E. (1993). Fundamentals of polymer solutions. Interactions of Surfactants with Polymers and Proteins.

[44] Wu X., Hou J., Li M., Wang J., Kaplan D.L., Lu S. (2012). Sodium dodecyl sulfate-induced rapid gelation of silk fibroin. Acta Biomater..

[45] Yang Y., Dicko C., Bain C.D., Gong Z., Jacobs R.M.J., Shao Z., Terry A.E., Vollrath F. (2012). Behavior of silk protein at the air–water interface. Soft Matter.

[46] Ishimuro Y., Ueberreiter K. (1980). The surface tension of poly(acrylic acid) in aqueous solution. Colloid Polym. Sci..

[47] Babak V.G., Skotnikova E.A., Lukina I.G., Pelletier S., Hubert P., Dellacherie E. (2000). Hydrophobically Associating Alginate Derivatives: Surface Tension Properties of Their Mixed Aqueous Solutions with Oppositely Charged Surfactants. J. Colloid Interface Sci..

[48] Taylor D.J.F., Thomas R.K., Penfold J. (2007). Polymer/surfactant interactions at the air/water interface. Adv. Colloid Interface Sci..

